# Supplementary material for: Prognosis and Immunological Characteristics of PGK1 in Lung Adenocarcinoma: A Systematic Analysis
Source: Cancers (Basel). 2022 Oct 25;14(21):5228. doi: 10.3390/cancers14215228 (PMC9653683; doi:10.3390/cancers14215228)
Supplement: Supplementary file 1 [file cancers-14-05228-s001.zip › cancers-1943060-supplementary.pdf]

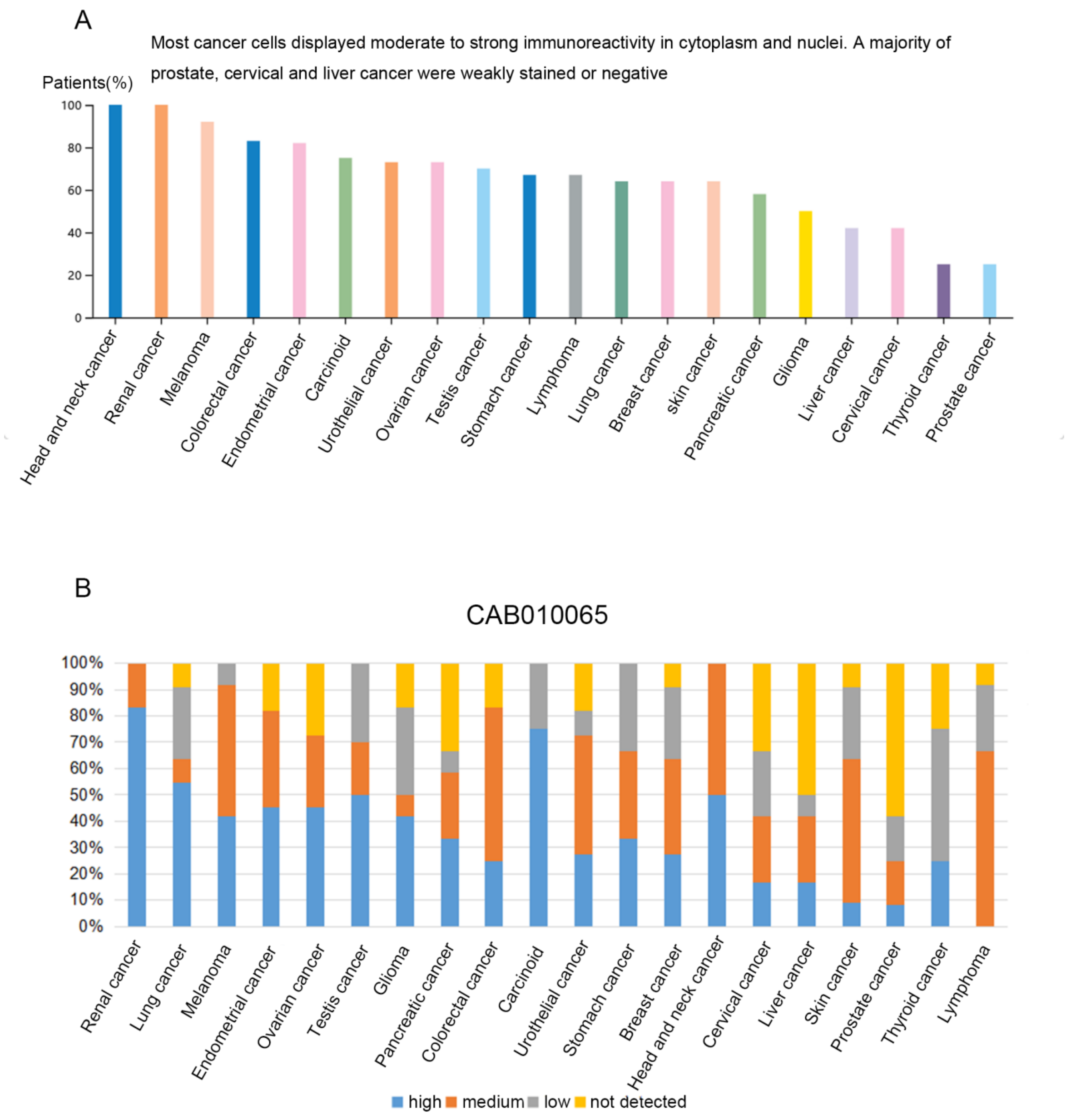

Figure S1: Expression of PGK1 in pan-cancer in HPA database.

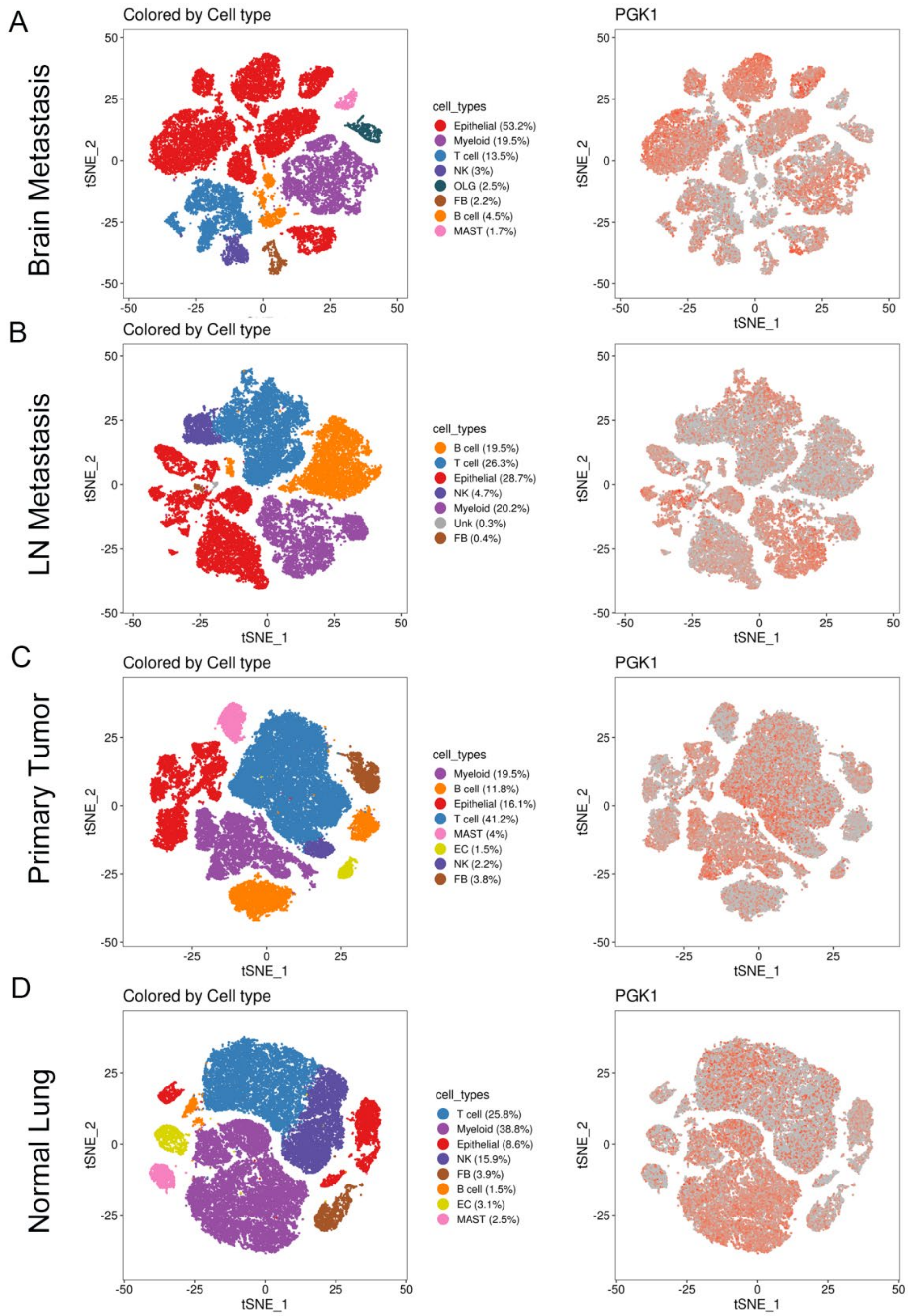

Figure S2: Expression of PGK1 at single cell level in GSE131907.

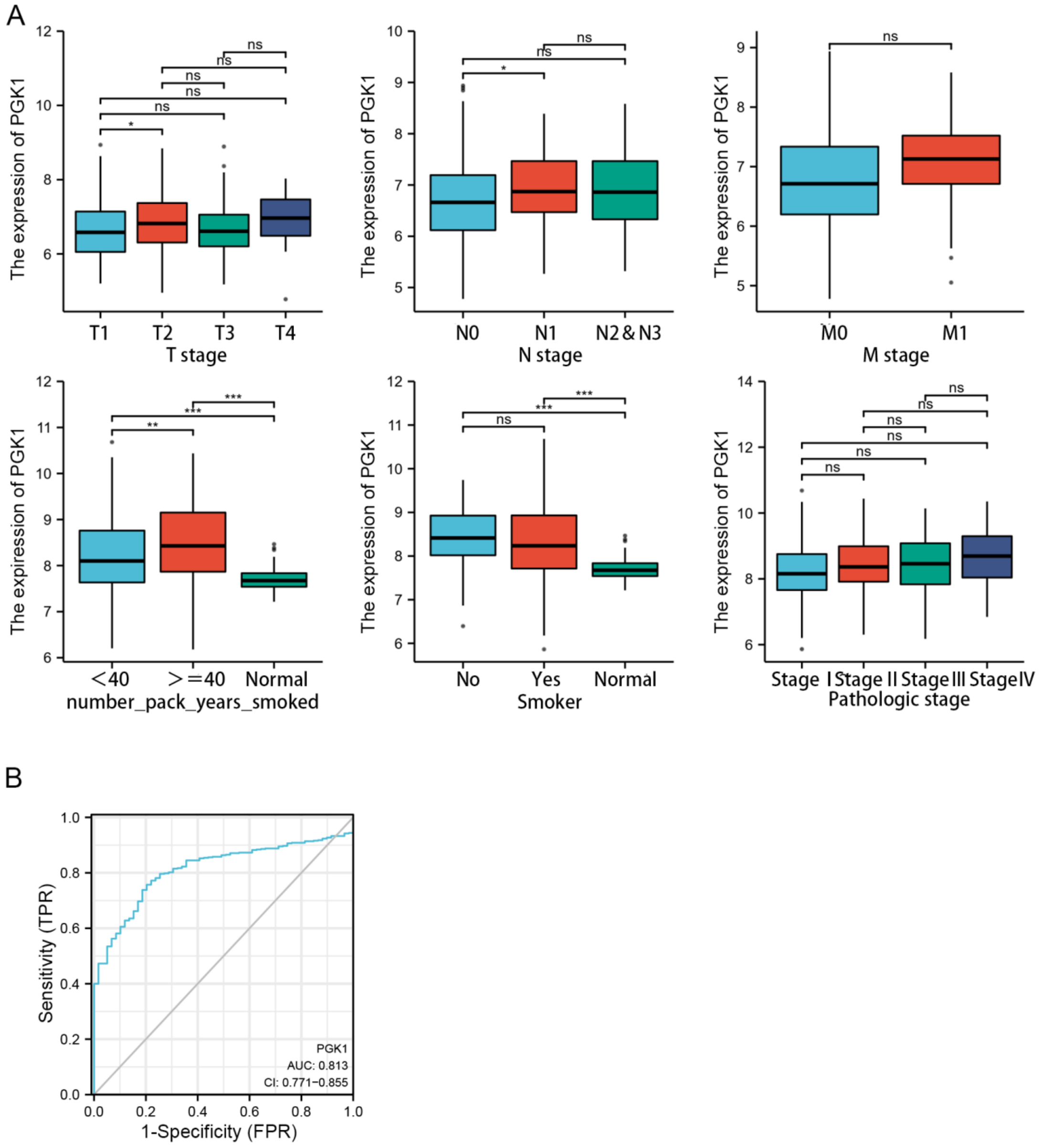

Figure S3: Correlation between PGK1 expression and clinical features of lung adenocarcinoma.

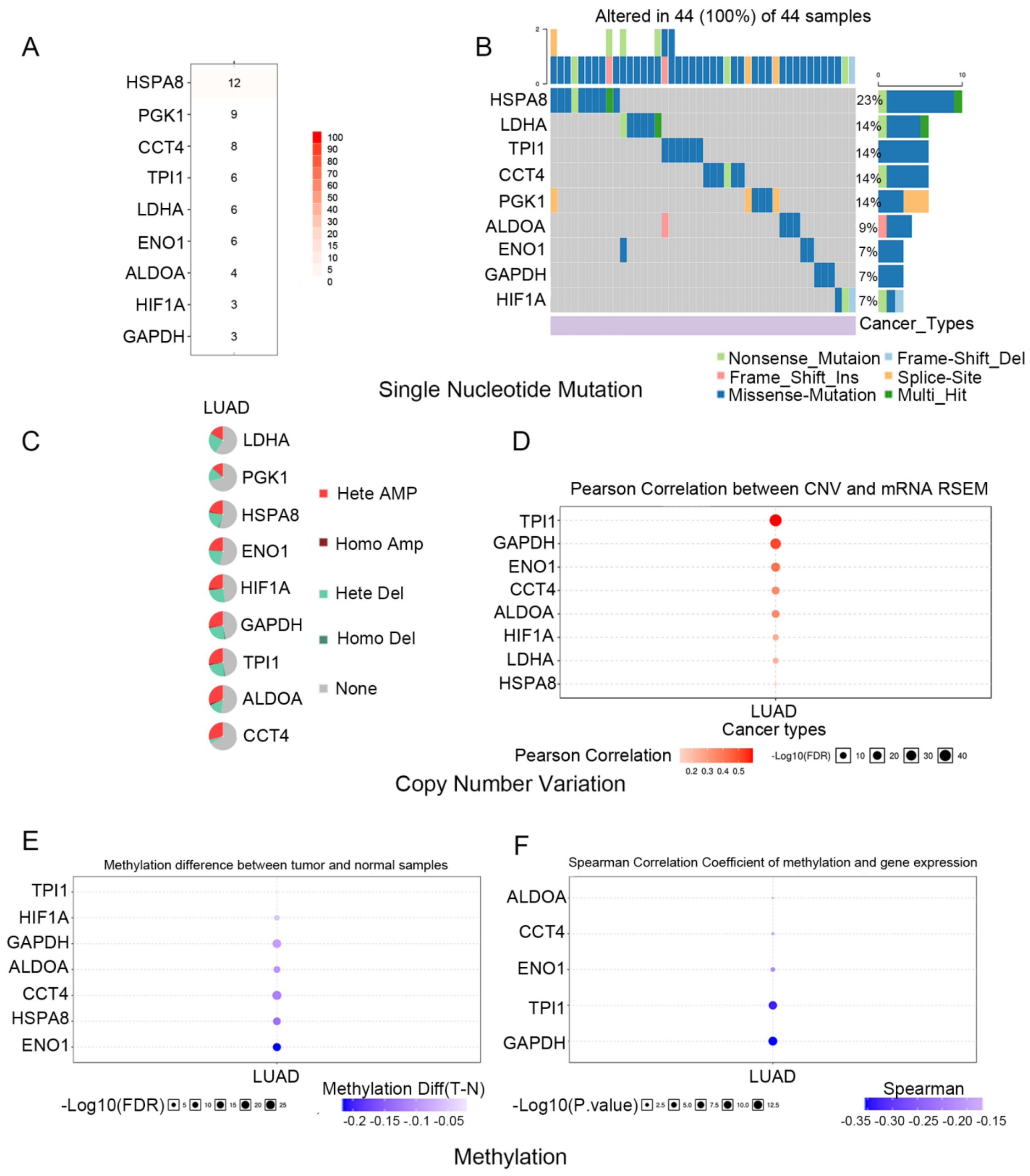

Figure S4: Genetic alteration analysis of PGK1 and its co-expressed genes

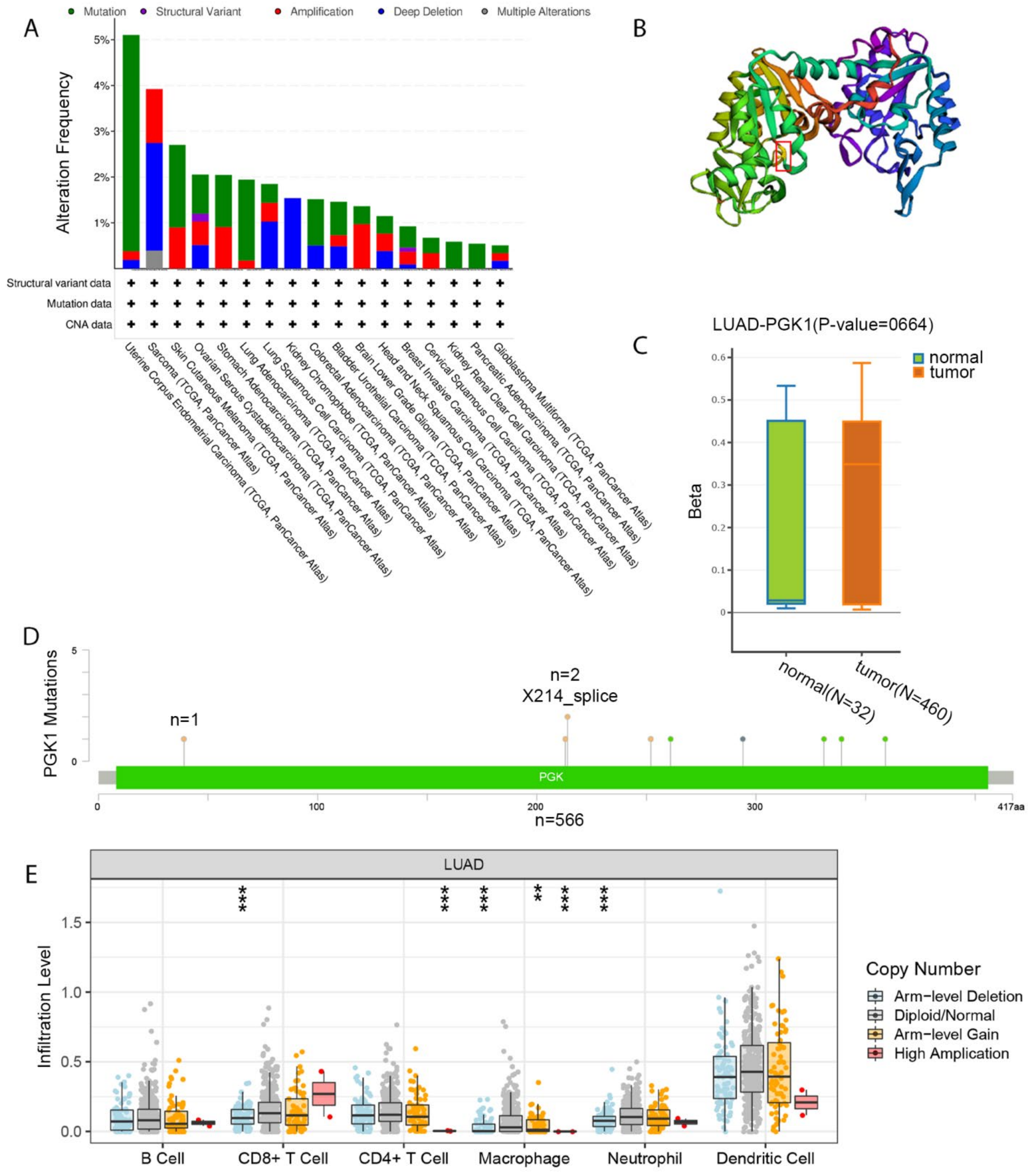

Figure S5: Analysis of PGK1 gene alteration in cbioportal and its effect on immune infiltration

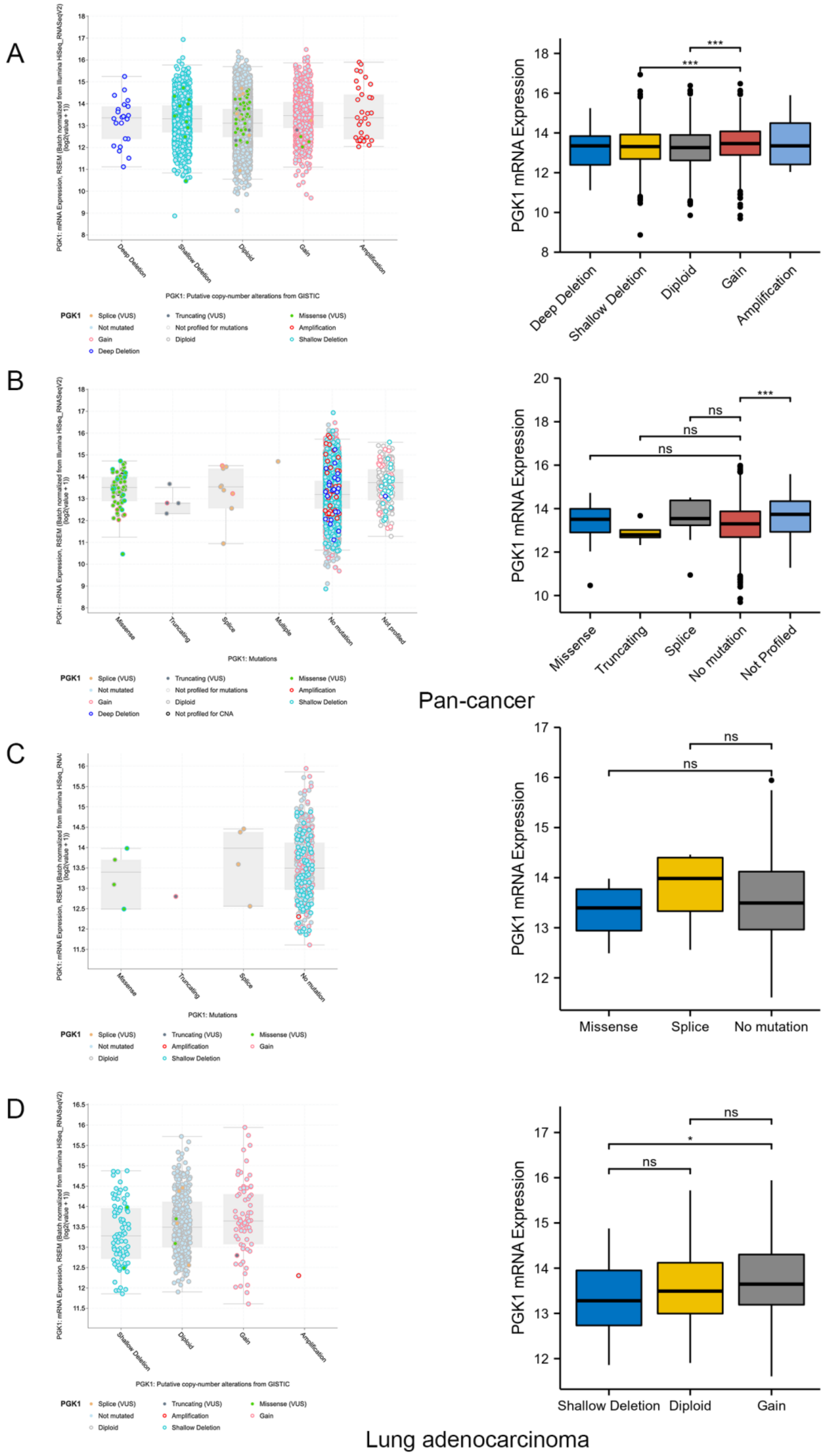

Figure S6: Effect of PGK1 gene alteration on mRNA expression of PGK1.

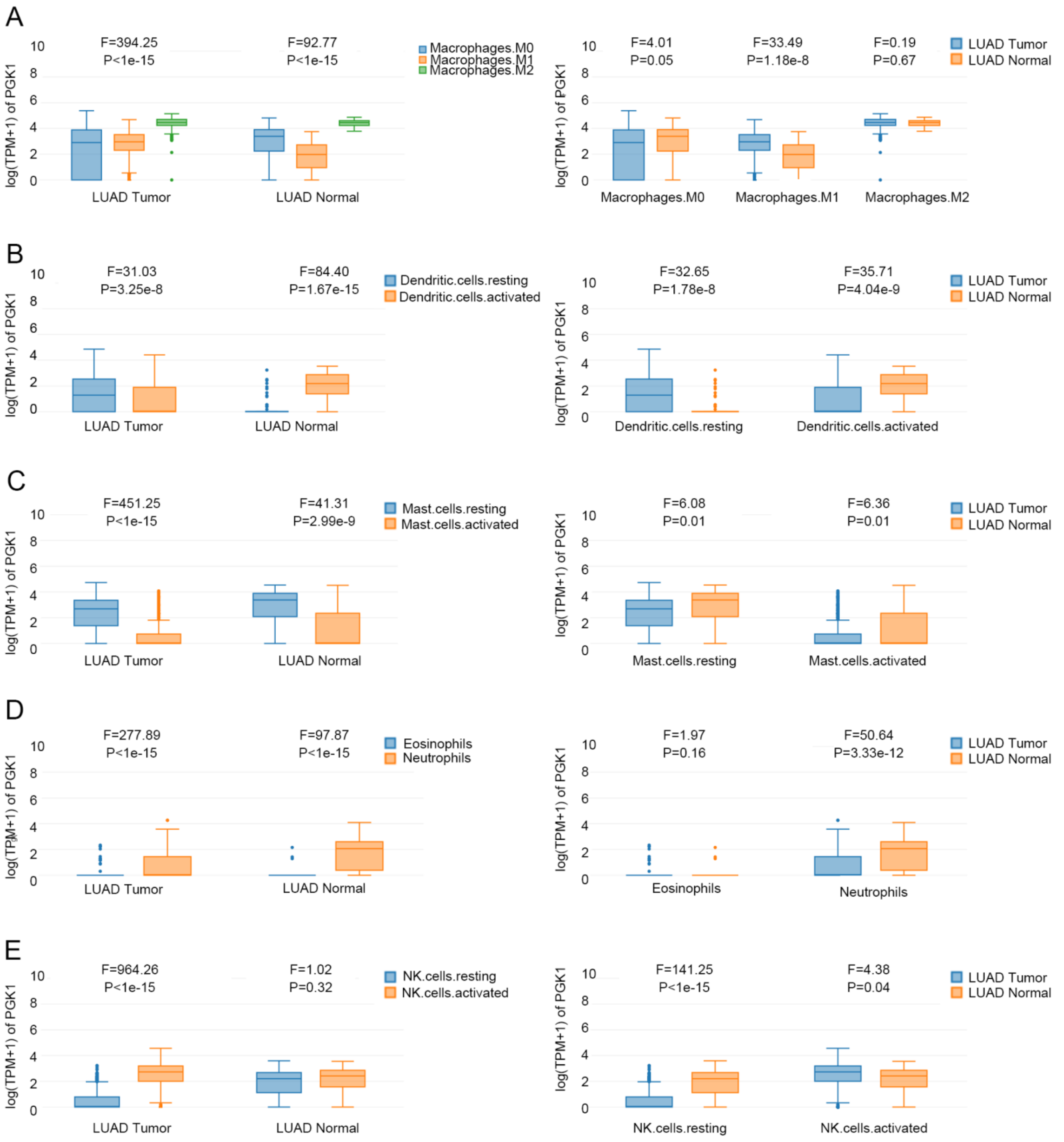

Figure S7. Effect of PGK1 expression on infiltration of various immune cells.

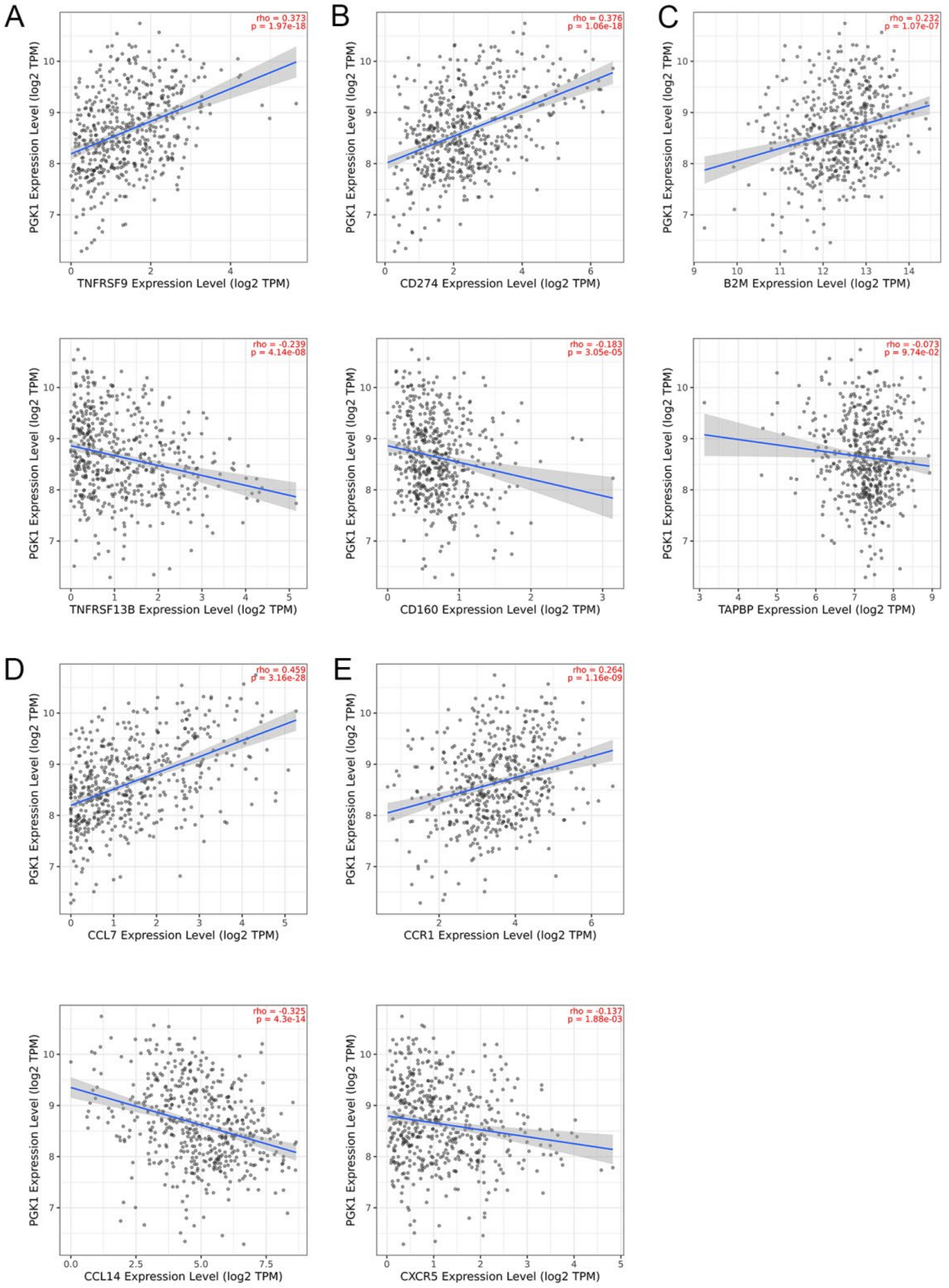

Figure S8: Correlation analysis between PGK1 and immune checkpoint in TIMER2 database.
